# Work–Family Conflict and Intention to Leave Among Nursing Professionals: A Cross-Sectional Study

**DOI:** 10.3390/healthcare14101382

**Published:** 2026-05-18

**Authors:** João Miguel Almeida Ventura-Silva, Olga Maria Pimenta Lopes Ribeiro, Marlene Patrícia Ribeiro, Ana da Conceição Alves Faria, Sónia Cristina da Costa Barros, Renata Cristina Gasparino, Clarissa Bohrer Silva, Elaine Cristina Novatzki Forte, Mattia Bozzetti, Nilüfer Demirsoy, Samuel Spiegelberg Zuge

**Affiliations:** 1 Northern Health School of the Portuguese Red Cross, 3720-126 Oliveira de Azeméis, Portugal; 2 RISE-Health, 4200-319 Porto, Portugal; olgaribeiro@esenf.pt; 3 RISE-Health, Nursing School, University of Porto, 4200-072 Porto, Portugal; 4 Local Health Unit Tâmega e Sousa, 4560-136 Penafiel, Portugal; marlene.ribeiro@ulsts.min-saude.pt; 5 Abel Salazar Institute of Biomedical Sciences, University of Porto, 4050-313 Porto, Portugal; 6 Department of Nursing, Santa Maria Higher School of Health, 4049-024 Porto, Portugal; ana.faria@santamariasaude.pt; 7 Local Health Unit São João, 4200-319 Porto, Portugal; sonia.barros@ulssjoao.min-saude.pt; 8 Faculty of Nursing, State University of Campinas, Campinas 13083-887, Brazil; grenata@unicamp.br; 9 Department of Nursing, Santa Catarina State University, Chapecó 88035-901, Brazil; clarissa.bohrer@udesc.br; 10 Department of Nursing, Federal University of Santa Catarina, Florianópolis 88040-900, Brazil; elaine.forte@ufsc.br; 11 Direction of Health Professions, Territorial Social Health Authority, 126100 Cremona, Italy; mattia.bozzetti@asst-cremona.it; 12 Faculty of Medicine, Eskisehir Osmangazi University, 26040 Eskisehir, Turkey; npoyraz@ogu.edu.tr; 13 Department of Nursing, Community University of the Chapecó Region, Chapecó 89809-900, Brazil; samuel.zuge@unochapeco.edu.br

**Keywords:** nurses, work–life balance, personnel turnover

## Abstract

**Background/Objectives**: Work–family conflict is a relevant psychosocial factor in nursing and may influence turnover intention. This study analyzed the association between work–family conflict and turnover intention among nursing professionals in Portugal. **Methods**: A cross-sectional study was conducted between April and July 2025 with 1097 nurses from a local health unit in northern Portugal. Data were collected using a sociodemographic and professional questionnaire, the Work–Family Conflict (WFC) and Family–Work Conflict (FWC) Scale, and the Turnover Intention Scale. Nonparametric tests, Spearman’s correlation, and linear regression models were used. **Results**: Participants were aged 23–66 years, with a median age of 42 years; most were women (84.0%) and held a bachelor’s degree (85.0%). Mean scores were 24.5 (SD = 7.7) for WFC, 13.2 (SD = 6.6) for FWC, and 34.8 (SD = 15.7) for turnover intention. Both conflict dimensions were positively correlated with turnover intention, with a stronger association for WFC (ρ = 0.347; *p* < 0.001) than for FWC (ρ = 0.186; *p* < 0.001). In the fully adjusted regression model, WFC remained positively associated with turnover intention (β = 0.383; SE = 0.062; t = 6.149; *p* < 0.001), whereas FWC was no longer significant. Adding job satisfaction, perceived work-related stress, and nurse manager support increased explanatory capacity. **Conclusions**: Work–family conflict, especially work-to-family interference, was associated with nurses’ turnover intention. These findings highlight the need for organizational strategies that reduce work demands, support work–life balance, and address broader occupational and psychosocial conditions.

## 1. Introduction

Nursing represents an essential professional category for ensuring the quality of care and the uninterrupted functioning of health services [1,2]. In highly demanding contexts, such as contemporary health systems, nursing professionals, especially leaders, are constantly challenged to simultaneously manage the quality and safety of care, continuity of care, and the sustainability of teams [3,4,5]. In the Portuguese context, this issue takes on particular relevance, since recent debates on the sustainability of the National Health Service, the aging of the workforce, and the retention of nurses have highlighted the need to understand the factors that influence these professionals’ decision to remain in the workforce [6,7].

In the Portuguese National Health Service, although the nursing workforce increased by 31% between 2010 and 2023, reaching approximately 51,000 professionals, one fifth of nurses are currently aged 55 years or older. In addition, it has been estimated that around 3900 full-time equivalent nurses would be needed to reduce reliance on overtime and temporary work [6]. These indicators reinforce the relevance of nurse retention in the Portuguese context.

Given the nature of nursing work, nurses are frequently exposed to long working hours, shift work and on-call duties, workload overload, high levels of responsibility, and intense emotional demands [8,9]. In addition, staff shortages further aggravate these challenges, requiring professionals to extend their working hours and take on additional responsibilities [10]. This scenario may increase exhaustion, disengagement, and turnover intention, with implications for workforce stability and care continuity [11,12]. In Portugal, this concern is reinforced by recent evidence highlighting the retention and stability of nursing teams as critical dimensions for organizational sustainability and for the responsiveness of health services [13].

Another issue related to the work of nursing professionals involves the tensions generated by work–family conflict, which arises when job demands and family responsibilities become incompatible [14,15,16]. This construct is recognized as bidirectional (interference of work with family and of family with work) and may arise from time pressures and accumulated strain, with relevant repercussions for well-being and the occupational experience [17,18]. In the case of nursing, scheduling patterns, shift work, and the intensity of emotional demands make this conflict especially salient, which justifies its analysis in nurse populations and also supports the relevance of assessing it in the Portuguese context [19,20].

Consequently, work–family conflict has been associated with increased perceived stress, emotional exhaustion, and burnout, as well as with reduced job satisfaction, engagement, and organizational commitment, elements that are central to remaining in employment and to the quality of care. This set of adverse effects may, in turn, increase turnover intention, understood as a proximal indicator of disengagement and staff turnover processes in nursing contexts [20,21,22].

Turnover intention may take different forms, depending on the scope adopted in the study: actual turnover, when the professional effectively leaves the institution; the intention to change sector/unit or to change jobs, often related to the search for more favorable conditions in terms of workload, support, and work environment; and the intention to leave the profession, a dimension that is especially relevant in contexts of chronic strain and perceived devaluation [9,22]. In this sense, turnover intention can be understood as an early sign of fragility in the occupational bond, with particular relevance in settings where nurse retention has become a strategic priority, as has recently been highlighted in Portugal [7].

Although turnover intention reflects a cognitive predisposition to leave the workplace or the profession [22], quiet quitting can be understood as a form of psychological and behavioral withdrawal in which professionals remain formally employed but reduce their emotional and discretionary investment in work. Thus, both phenomena reflect a weakening of the occupational bond and may share psychosocial antecedents, such as work–family conflict, stress, burnout, and perceived lack of organizational support [21,23,24,25]. This theoretical connection supports the inclusion of the present study within a broader research project on quiet quitting, burnout, and work–family conflict in nursing.

Perceived organizational support and nurse manager support are often described as protective factors: they may reduce work–family conflict and/or weaken its association with turnover intention. Accordingly, practices such as flexibility, fairness in scheduling, communication, recognition, and instrumental support become especially relevant as strategies for retention and the promotion of well-being [23,24,25,26,27].

Despite advances in knowledge, gaps remain that limit the robustness and applicability of the findings, including an uneven body of evidence across countries and care settings, as well as the need for more explanatory models that test mechanisms and conditions (mediations and moderations) [22,28]. Moreover, in the European context, and particularly in Portugal, it remains important to produce context-specific evidence that makes it possible to understand how organizational, professional, and work–family balance factors interact in the retention of nurses [6,7,13]. In light of this context, the present study aims to analyze the association between work–family conflict and turnover intention among nursing professionals in Portugal. By focusing on this context, the study seeks to contribute to evidence-informed strategies for nurse retention and work–family balance.

## 2. Materials and Methods

### 2.1. Study Design

The study adopted a quantitative, observational, analytical, and cross-sectional design, with reporting aligned to the Strengthening the Reporting of Observational Studies in Epidemiology (STROBE) Statement [29].

### 2.2. Setting and Participants

Data collection was conducted from April to July 2025 at a local health unit located in northern Portugal. These units are public institutions designed to deliver integrated primary and hospital care, based on the coordination of care levels across different settings to prioritize citizen-centered interventions.

The local health unit encompasses two clusters of health centers dedicated to primary care, along with two hospitals. The central hospital serves as a national reference center for healthcare provision, training of health professionals, research, and innovation. The secondary hospital functions as a supporting facility, offering outpatient and rehabilitation services.

Potential participants included nurses working in primary healthcare settings and in hospital departments of medicine, surgery, women’s and children’s health, and emergency services. The inclusion criteria were nurses working in the local health unit during the data collection period, regardless of employment status, professional category, or care setting, who had at least six months of professional experience. The exclusion criteria were fewer than six months of professional experience, prolonged absence from work due to leave, illness, or other reasons during data collection, absence of valid informed consent, or insufficient questionnaire completion. Nurses with fewer than six months of experience were excluded to reduce the influence of the initial integration, orientation, and adaptation period, during which perceptions of work–family conflict, stress, and turnover intention may primarily reflect professional socialization rather than a more stabilized work experience within the service.

Participants were approached in person by the researchers in the different care settings, after prior scheduling with the services and in collaboration with nurse managers. Recruitment followed a census-based approach, as all eligible nurses available during the data collection period were invited to participate.

After applying the inclusion and exclusion criteria, 2585 individuals were eligible at the local health unit. A total of 1141 questionnaires were returned, of which 44 were excluded due to incomplete responses. Thus, the final sample comprised 1097 participants, corresponding to a response rate of 42.4%.

### 2.3. Data Collection Instruments

Data collection employed a two-section questionnaire. The initial section comprised questions intended to characterize participants’ sociodemographic, professional, and academic profiles. These encompassed gender, marital status, age, total years of professional experience, current job tenure, employment contract type, professional category, and work schedule.

In the second part of the questionnaire, the Work–Family Conflict (WFC) and Family–Work Conflict (FWC) Scale [19] and the Turnover Intention Scale [30] were administered.

#### 2.3.1. Work–Family Conflict (WFC) and Family–Work Conflict (FWC) Scale

The Work–Family Conflict Scale was originally developed by Netemeyer et al. (1996) to assess bidirectional conflict between work and family domains, specifically the interference of work with family life and the interference of family with work responsibilities [31].

The instrument comprises two subscales corresponding to each direction of conflict: WFC and FWC. Each subscale contains five items, resulting in a total of 10 items.

Participants indicate their level of agreement with each statement using a seven-point Likert scale ranging from 1 (strongly disagree) to 7 (strongly agree).

According to Netemeyer et al. (1996) [31], the overall level of work–family conflict can be calculated by summing the scores across all 10 items, yielding a total score ranging from 10 to 70, with higher scores indicating greater levels of conflict between professional and family roles. In addition, separate scores can be computed for each dimension. The WFC score is obtained by summing the first five items, reflecting the perceived interference of work with family life, while the FWC score is obtained by summing the last five items, reflecting the perceived interference of family responsibilities with work. Each subscale score ranges from 5 to 35, with higher values indicating greater perceived interference between the two domains [31].

The instrument was later adapted and validated for the Portuguese context by Simães et al. (2019) [19], who preserved the original structure of the scale. The authors reported satisfactory internal consistency, with a global Cronbach’s alpha of 0.88. Regarding the subscales, Cronbach’s alpha values were 0.91 for the WFC subscale and 0.85 for the FWC subscale, indicating good reliability of the instrument for assessing bidirectional work–family conflict in the Portuguese population [19].

#### 2.3.2. Turnover Intention Scale

The Anticipated Turnover Scale (ATS) was originally developed by Hinshaw and Atwood (1984) [32] to assess nurses’ intention to leave their current job and/or the nursing profession. In the original study, the instrument demonstrated good psychometric properties, with an internal consistency of 0.84 according to Cronbach’s alpha, and factor analysis identifying two factors that explained 54.9% of the variance [32].

For the Portuguese cultural context, the scale was adapted and validated by Sul and Lucas (2020) [30]. In this version, the instrument comprises 10 items rated on a Likert-type scale ranging from 1 (strongly disagree) to 7 (strongly agree), resulting in a total score that can vary from 10 to 70, with higher scores indicating a greater risk of turnover. In the study conducted by Sul and Lucas (2020), the Portuguese version of the ATS showed excellent internal consistency, with a Cronbach’s alpha of 0.910 [30].

### 2.4. Data Collection Procedures

Following prior scheduling adjusted to the professionals’ availability, the researchers visited the various care settings. In collaboration with the nurse managers, they provided each participant with information about the study’s objectives and procedures, the Free and Informed Consent Form, and the data collection instrument. Participants expressed their agreement by signing the consent form. To reduce the risk of social desirability bias, participants were instructed to complete the instrument privately and voluntarily, without the direct presence of nurse managers at the time of completion. To preserve anonymity, two unmarked envelopes were provided, in which the signed consent form and the completed questionnaire were deposited separately. Confidentiality and anonymity were ensured in the use and disclosure of the collected data.

### 2.5. Data Analysis

Data were organized and analyzed using R software, version 4.4.1. Categorical variables were presented as absolute and relative frequencies, whereas continuous variables were described using means, standard deviations, and minimum and maximum values. The WFC, FWC, and ATS scores were analyzed as continuous variables, without applying cut-off points to categorize them into low, moderate, or high levels. The categories used for job satisfaction, perceived work-related stress, and perceived support/supervision from the nurse manager were predefined in the sociodemographic and professional questionnaire and used for descriptive and comparative purposes. The internal consistency of the instruments was assessed using Cronbach’s alpha.

Initially, participants were characterized according to their sociodemographic and professional profile, followed by the descriptive analysis of work–family conflict, family–work conflict, and turnover intention scores. The distribution of continuous variables was assessed using the Shapiro–Wilk test. Given the absence of normality, nonparametric tests were adopted for comparative analyses. The Mann–Whitney U test was used for comparisons between two groups, and the Kruskal–Wallis test was used for comparisons among three or more groups. When statistically significant differences were identified in multiple-group comparisons, Dunn’s post hoc test with Bonferroni correction was applied.

The association between the dimensions of work–family conflict and turnover intention was examined using Spearman’s correlation coefficient. Subsequently, linear regression models were fitted, considering turnover intention as the dependent variable and the work–family conflict and family–work conflict dimensions as independent variables. Three models were estimated: an unadjusted model; a model adjusted for sex, age, marital status, and number of dependent children; and an additional model adjusted for occupational variables, specifically job satisfaction, perceived work-related stress, and perceived support and supervision from the nurse manager. Results were reported as beta coefficients, standard errors, t values, *p* values, and adjusted coefficients of determination. Although the continuous variables did not present a normal distribution according to the Shapiro–Wilk test, linear regression was used to estimate adjusted associations between the dimensions of work–family conflict and turnover intention. This decision considered that, in linear regression, the normality assumption is especially relevant to the model residuals rather than to the isolated distribution of the independent or dependent variables. Therefore, model interpretation considered the assumptions of linearity, homoscedasticity, normality of residuals, and absence of multicollinearity, as well as the large sample size of the study. A significance level of 5% (*p* < 0.05) was adopted for all analyses.

### 2.6. Ethical Approval and Informed Consent

The study was approved by the Health Research Ethics Committee (opinion no. 51-2025), followed by authorization for data collection granted by the hospital’s Board of Directors. Nurses who agreed to participate signed the informed consent form, and confidentiality and anonymity were preserved. Participants placed their completed questionnaires in unmarked envelopes, which were then collected by the researchers. The present study is part of a wider research project entitled “Quiet Quitting na Enfermagem: Como o Burnout e os Conflitos Trabalho-Família Influenciam a Intenção de Sair”.

## 3. Results

A total of 1097 nurses participated in the study, aged 23 to 66 years, with a median age of 42 years. The sample showed a predominance of Generation Y professionals (52.2%), female participants (84.0%), those with a partner (65.0%), and those holding a bachelor’s degree (85.0%) (Table 1).

In the comparative analyses, statistically significant differences were observed in WFC according to gender, age group, professional category, job satisfaction, perceived work-related stress, perceived support and supervision from the nurse manager, organizational attachment, and intention to remain in the institution. For FWC, statistically significant differences were identified according to age group, marital status, professional category, job satisfaction, perceived work-related stress, perceived support and supervision from the nurse manager, and organizational attachment. Turnover intention differed significantly according to age group, marital status, professional category, job satisfaction, perceived work-related stress, perceived support and supervision from the nurse manager, organizational attachment, and intention to remain in the institution (Table 2).

The descriptive results of the scales indicated higher mean scores for WFC compared with FWC. WFC had a mean score of 24.5 (SD = 7.7), whereas FWC had a mean score of 13.2 (SD = 6.6). Finally, turnover intention had a mean score of 34.8 (SD = 15.7). In addition, all measures showed satisfactory internal consistency, with Cronbach’s alpha values ranging from 0.82 to 0.92 (Table 3).

Spearman’s correlation analysis showed a positive and statistically significant association between both conflict dimensions and turnover intention. A moderate positive correlation was observed between work–family conflict and turnover intention (ρ = 0.347; *p* < 0.001), indicating that higher levels of work interference with family life were associated with a greater intention to leave. Likewise, a positive, although weaker, correlation was identified between family–work conflict and turnover intention (ρ = 0.186; *p* < 0.001), suggesting that interference of family demands with work was also associated with increased turnover intention, albeit with lower magnitude. Overall, these findings indicate that both conflict dimensions are related to turnover intention, with a stronger association observed for work–family conflict (Table 4).

The regression models consistently showed that WFC was positively associated with turnover intention, whereas FWC did not show a statistically significant association. In Model A, the unadjusted model, WFC was associated with turnover intention (β = 0.677; SE = 0.064; t = 10.570; *p* < 0.001), whereas FWC was not significant, and the model explained 11.7% of the variance in the outcome. In Model B, adjusted for sex, age, marital status, and number of dependent children, WFC remained significantly associated with turnover intention (β = 0.649; SE = 0.065; t = 9.993; *p* < 0.001), whereas FWC remained non-significant, with an adjusted R^2^ of 0.121. Finally, in Model C, adjusted for job satisfaction, perceived work-related stress, and perceived support and supervision from the nurse manager, WFC maintained a positive and significant association with turnover intention (β = 0.383; SE = 0.062; t = 6.149; *p* < 0.001), whereas FWC remained statistically non-significant. This model showed the greatest explanatory power, with an adjusted R^2^ of 0.276 (Table 4).

The increase in adjusted R^2^ from Model B to Model C indicates that the inclusion of occupational variables added relevant explanatory capacity to the model. Therefore, although WFC remained associated with turnover intention, job satisfaction, perceived work-related stress, and perceived support and supervision from the nurse manager should also be considered important factors in interpreting this outcome.

## 4. Discussion

WFC showed higher values than FWC, indicating that, among nurses, the interference of work in family life tends to be more significant than the reverse. This pattern is plausible in the context of nursing, given that the profession involves shift work, demanding working hours, caregiving pressure, emotional intensity, and reduced predictability of working time. Consequently, work demands tend to invade the family sphere more easily than family demands interfere with professional performance. This asymmetry also helps to understand why WFC showed a stronger association with the intention to leave than FWC, suggesting that, for these professionals, the intrusion of work into personal life may carry more weight in the decision to consider leaving the institution [21,33].

Correlational analysis and regression models reinforce this interpretation. Although both dimensions of conflict were positively associated with the intention to leave, only WFC remained significantly associated in the final model. This result suggests that the perception that work compromises family life may be more determinant in the formation of thoughts of leaving than family interference in work. Recent literature supports this interpretation, showing not only the association between work–family conflict and turnover intention, but also that this relationship can be mediated by psychological and emotional resources, which helps to explain the persistence of the WFC effect even when other variables are considered [34,35].

The fact that FWC did not remain significant in the adjusted models does not mean, however, that this dimension is irrelevant. On the contrary, the results suggest that its effect may be more contextual, indirect, or dependent on specific characteristics of certain groups. This reading is consistent with the study by Hu et al., which identified work–family conflict as a common factor in the intention to leave among different generations of nurses, but showed that family–work conflict took on particular relevance in Generation Y. Similarly, the synthesis by Figueiredo et al. highlights that work–life balance and the practice environment do not affect all groups in the same way, with vulnerability being especially evident in younger professionals [11,36].

A non-significant association for FWC in the final model may be partly explained by the way family demands are managed in the Portuguese sociocultural context. Although Portugal is characterized by a dual-earner model, domestic and caregiving responsibilities may still be unequally distributed and strongly shaped by gender roles, particularly in a predominantly female profession such as nursing [37]. In this context, family demands may be managed through private arrangements and informal support networks, making FWC less likely to emerge as an independent predictor of turnover intention. By contrast, WFC reflects institutional demands, such as shift work, unpredictable schedules, and emotional intensity, over which professionals may have less control, thereby making work interference with family life more salient in the decision to consider leaving [38].

Even though FWC was not statistically significant in the final model, it should not be dismissed from an organizational perspective. In nursing teams, especially in large workforces, family-related pressures may still affect specific subgroups, such as nurses with young children, single-parent families, informal caregivers, or professionals with limited family support, and contribute indirectly to stress, lower well-being, and vulnerability to turnover. Therefore, the interpretation of these findings should consider not only statistical significance but also their relevance for workforce management and nurse retention.

Age proved to be, in fact, an important element in understanding these differences. Generation Y presented the highest values of WFC, FWC, and intention to leave, suggesting greater vulnerability in this group. This trend may reflect a phase of life marked by the simultaneous accumulation of professional, marital, and parental demands, as well as higher expectations regarding the balance between professional and personal life. Post hoc analyses also showed that age differences were not homogeneous across all groups, concentrating mainly in Generation Y. This interpretation is consistent with studies that link work–life balance, work stress, and work–family conflict with the intention to leave, especially when job satisfaction is affected [39,40].

The greater vulnerability of Generation Y may be understood through the convergence of career stage and life cycle demands. Professionals in this group are often in a period of career consolidation while simultaneously facing family formation, parenthood, and caregiving responsibilities. Studies indicate that professionals with young children tend to report higher levels of work–family conflict, as caregiving demands in this stage are intense and less flexible [41]. In addition, younger nurses may experience greater occupational stress related to the consolidation of clinical skills and adaptation to organizational culture, while also placing greater value on quality of life and work–life balance than on traditional organizational loyalty [42,43].

Another key aspect concerns the role of occupational variables included in the final model. The fact that WFC remained significantly associated with the intention to leave even after adjusting for job satisfaction, perceived work stress, and nurse manager support/supervision reinforces the robustness of this association. At the same time, the increased explanatory power of the model shows that the intention to leave depends not only on work–family conflict but also on the psychosocial and organizational conditions of the work. This interpretation is consistent with evidence showing that work-related stress is associated with the intention to leave and burnout, and that the perceived competence of the nurse manager is related to greater job satisfaction and less intention to leave [44,45].

Along the same lines, nurses with lower job satisfaction, higher perceived stress, lower perceived support and supervision from the nurse manager, and lower organizational attachment showed higher values of WFC, FWC, and intention to leave. This pattern confirms that the intention to leave should be interpreted not only as an individual response to role conflict, but also as an expression of the quality of the work context. More recent evidence shows that social support and perceived organizational support are negatively associated with turnover intention and that this effect may occur through greater psychological resilience and less fatigue associated with organizational change [24,46].

The increased explanatory power of the final model also has important conceptual implications. It suggests that the intention to leave is a multifactorial phenomenon, in which work–family conflict is articulated with the practice environment, leadership, organizational support, and available resources. This reading is consistent with studies that show a moderate negative correlation between organizational support and intention to leave, as well as a robust relationship between job embeddedness and lower turnover intention [47,48,49]. Thus, although WFC is an important predictor, its impact should be understood within the broader framework of institutional conditions that can exacerbate or mitigate the experience of conflict and the predisposition to leave the organization [23,50].

From a practical and managerial point of view, these findings suggest that retention strategies should prioritize reducing work interference in family life. This implies acting on the organization of work and schedules, promoting greater predictability, reducing workload and occupational stress, and strengthening the support provided by the nurse manager. It also seems relevant to develop strategies that are more sensitive to generational profiles, especially for Generation Y professionals, who have emerged as a particularly vulnerable group. This interpretation is particularly pertinent in the Portuguese context, where nurses’ intention to remain in the NHS is related to overall satisfaction, work–life balance, schedule predictability, and professional development opportunities; similarly, recent studies show that transformational leadership, career growth, well-being at work, and work–life balance favor retention [1,7].

Concrete managerial strategies may include participatory schedule planning, self-scheduling models, greater predictability of shifts, limits on last-minute schedule changes, and monitoring of overtime hours. These measures may increase nurses’ perceived control over working time and facilitate the reconciliation of professional and family responsibilities [51]. In addition, institutions should consider formal psychological support and stress management programs, including emotional debriefing and burnout prevention initiatives, as well as training nurse managers in family-supportive leadership behaviors [52,53].

The relevance of schedule predictability and work organization is also supported by evidence showing that more predictable work schedules and organized nursing care are associated with greater job satisfaction and better quality of life [21,54]. Therefore, interventions aimed at improving the regularity of schedules and the organization of nursing work may contribute to intermediate outcomes that support retention, even if their direct effect on turnover intention is not immediate.

This study has some limitations. Cross-sectional design does not allow causal relationships to be established, and the use of self-report measures may introduce social desirability bias and common variance of the method. In addition, the sample was drawn from a single local health unit, which may limit the generalizability of the findings to other regions, private institutions, or different organizational models. The distribution by sex was also unbalanced, reflecting the feminized composition of the nursing profession, but potentially limiting robust comparisons between men and women. Furthermore, the absence of longitudinal data prevents assessing the temporal evolution of turnover intention or confirming whether this intention translates into actual turnover. Still, the sample size, the satisfactory internal consistency of the instruments, and the coherence between descriptive, correlational, and multivariate analyses reinforce the credibility of the findings.

Future research should consider multicenter and longitudinal designs, including different Portuguese regions and European contexts, as well as the testing of mediation and moderation models integrating burnout, quiet quitting, organizational support, family support, leadership, and caregiving responsibilities. It would also be relevant to analyze subgroups with parental or caregiving responsibilities and to combine self-reported measures with organizational indicators, such as absenteeism, actual turnover, and overtime hours.

Work–family conflict was more pronounced than family–work conflict and proved to be more relevant in explaining the intention to leave among nurses. This association remained even after controlling for relevant occupational variables, which reinforces its robustness, but the final model also showed that the intention to leave depends on the psychosocial and organizational conditions of the work. Thus, promoting more sustainable practice environments, with less stress, greater support from management, and a better work–family balance, may constitute an important strategy for retaining nurses.

## 5. Conclusions

This study showed that work–family conflict was associated with the intention to leave among nursing professionals. The work–family dimension showed the most consistent association with the outcome, remaining significant even after adjustment, while family–work conflict did not remain independently associated. These findings indicate that the intention to leave is more strongly related to the interference of work demands in family life, especially when compared to the interference of family demands in work.

The results reinforce the importance of institutional actions aimed at reducing work–family conflict, with the potential to contribute to the retention of nurses and the sustainability of nursing teams. Concrete interventions may include flexible and predictable schedules, participatory planning of work shifts, training of nurse managers focused on work–life balance, and institutional psychological support programs. In the Portuguese NHS context, where nurse retention and team sustainability are strategic priorities, such measures may help reduce turnover intention and strengthen workforce stability. Although these conclusions are grounded in the Portuguese context, they may also be relevant to other European health systems facing similar challenges related to workforce aging, professional shortages, care pressure, and nurse retention.

## Figures and Tables

**Table 1:** Sociodemographic and professional characteristics of the participants (*n* = 1097).

Variable	*n* (%)
Gender	
Male	175 (16.0)
Female	922 (84.0)
Age	
Generation Z (23–29 years)	125 (11.4)
Generation Y (30–45 years)	584 (53.2)
Generation X (46–61 years)	372 (33.9)
Baby Boomers (62–66 years)	16 (1.5)
Marital status	
Without a partner	315 (28.7)
With a partner	782 (71.3)
Educational qualification	
Bachelor’s degree/Graduation	932 (85.0)
Master’s degree	165 (15.0)
Condition of exercise of the profession	
Nurse	696 (63.4)
Specialist nurse	370 (33.7)
Manager nurse	31 (2.8)
Job satisfaction	
Low/Moderate	881 (80.3)
High	216 (19.7)
Perceived work-related stress	
Low/Moderate	515 (47.0)
High	582 (53.0)
Perceived support and supervision from the nurse manager	
Low/Moderate	901 (82.1)
High	196 (17.9)

Note: n = number of participants; % = percentage.

**Table 2:** Scores of work–family conflict, family–work conflict, and turnover intention according to sociodemographic and professional variables (*n* = 1097).

	WFC		FWC		ATS	
Variable	Mean (DP)	*p*	Mean (DP)	*p*	Mean (SD)	*p*
Gender		0.01		0.13		0.30
Male	25.8 (7.5)		13.7 (6.5)		35.9 (15.9)	
Female	24.2 (7.7)		13.0 (6.6)		34.6 (15.7)	
Age		<0.00		<0.00		<0.00
Generation Z (23–29 years) ^a^	24.1(8.0) ^b^		11.4 (6.2) ^b^		32.1 (16.3)	
Generation Y (30–45 years) ^b^	25.8 (6.9) ^ac^		14.2 (6.7) ^ac^		37.5 (15.6) ^c^	
Generation X (46–61 years) ^c^	22.6 (8.2) ^b^		12.1 (6.2) ^b^		31.5 (15.1) ^b^	
Baby Boomers (62–66 years) ^d^	19.9 (10.5)		11.2 (6.0)		31.5 (13.0)	
Marital status		0.48		0.04		0.03
Without a partner	24.6 (7.7)		12.6 (6.6)		36.2 (15.8)	
With a partner	24.4 (7.7)		13.4 (6.6)		34.0 (15.6)	
Educational qualification		0.07		0.68		0.95
Bachelor’s degree/Graduation	24.6 (7.7)		13.2 (6.6)		34.8 (15.8)	
Master’s degree	23.5 (7.8)		13.1 (6.9)		34.8 (15.4)	
Condition of exercise of the profession		0.00		0.03		0.00
Nurse ^a^	25.1 (7.4) ^bc^		13.5 (6.6) ^c^		35.3 (16.1) ^c^	
Specialist nurse ^b^	23.5 (8.1) ^a^		12.7 (6.6)		34.6 (15.1) ^c^	
Manager nurse ^c^	21.3 (8.5) ^a^		11.5 (5.9) ^a^		26.1 (11.8) ^ab^	
Job satisfaction		<0.00		<0.00		<0.00
Low/Moderate	25.4 (7.3)		13.7 (6.6)		37.4 (15.4)	
High	20.4 (8.1)		11.1 (6.3)		24.4 (12.5)	
Perceived work-related stress		<0.00		<0.00		<0.00
Low/Moderate	22.1 (8.1)		12.5 (6.6)		30.2 (14.2)	
High	26.6 (6.7)		13.8 (6.6)		38.9 (15.9)	
Perceived support and supervision from the nurse manager		<0.00		<0.00		<0.00
Low/Moderate	24.9 (7.5)		13.4 (6.6)		36.7 (15.7)	
High	22.4 (8.3)		12.0 (6.6)		25.9 (12.5)	
I feel attached to the organization and committed to remaining in it		<0.00		<0.00		<0.00
Yes	23.1 (7.8)		12.5 (6.5)		30.1 (14.3)	
No	27.1 (6.7)		14.4 (6.7)		43.9 (14.4)	
Intention to remain in the institution		<0.00		0.27		<0.00
Remain permanently	23.4 (7.8)		12.9 (6.5)		28.1 (13.8)	
Remain for one more year	25.9 (7.3)		13.5 (6.8)		43.9 (13.4)	

Note: SD = Standard deviation; *p*-value; WFC = Work-to-Family Conflict; FWC = Family–Work Conflict; ATS = Anticipated Turnover Scale. Superscript letters indicate statistically significant differences in Dunn’s post hoc comparisons with Bonferroni correction, performed after the Kruskal–Wallis test. Groups that do not share the same letter differ significantly from each other.

**Table 3:** Descriptive statistics of the instruments and their dimensions (mean, standard deviation, and minimum–maximum values) (*n* = 1097).

Variable	Mean (SD)	Minimum; Maximum	Cronbach’s
Work–Family Conflict and Family–Work Conflict Scale			
Work-to-Family Conflict	24.5 (7.7)	5; 35	0.92
Family–Work Conflict	13.2 (6.6)	5; 35	0.82
Turnover Intention Scale	34.8 (15.7)	10; 70	0.91

Note: SD = Standard deviation.

**Table 4:** Regression models examining the association between work–family conflict dimensions and turnover intention (*n* = 1097).

Model A				
Predictor	β	SE	*t*	*p*
WFC	0.677	0.064	10.570	<0.001
FWC	0.061	0.075	0.820	0.412
Model fit: R^2^ = 0.117				
Model B				
Predictor	β	SE	*t*	*p*
WFC	0.649	0.065	9.993	<0.001
FWC	0.093	0.076	1.221	0.222
Adjusted for: sex, age, marital status, number of dependent children.				
Model fit: Adjusted R^2^ = 0.121				
Model C				
Predictor	β	SE	*t*	*p*
WFC	0.383	0.062	6.149	<0.001
FWC	0.071	0.068	1.046	0.296
Adjusted for: Job satisfaction, perceived work-related stress, perceived support and supervision from the nurse manager.				
Model fit: Adjusted R^2^ = 0.276				

Note: WFC = Work-to-Family Conflict; FWC = Family–Work Conflict; β = regression coefficient; SE = standard error; *t* = *t*-test statistic; *p* = *p*-value.

## Data Availability

The data presented in this study is available upon request from the corresponding author. The data are not publicly available due to ethical restrictions.
